# Bridging the anatomical gap: evolutionary conservation of genetic mechanisms in corpus callosum disorders across human, mouse, and zebrafish

**DOI:** 10.3389/fnmol.2026.1823713

**Published:** 2026-05-07

**Authors:** Ayushma, Priyanka Prakash Srivastava, Shilpi Minocha

**Affiliations:** Kusuma School of Biological Sciences, Indian Institute of Technology Delhi, Hauz Khas, New Delhi, India

**Keywords:** agenesis of the corpus callosum, axon guidance, comparative biology, corpus callosum, mouse models, neurodevelopmental disorders, zebrafish

## Abstract

The Corpus Callosum (CC) is the largest white matter structure in the placental mammalian brain, facilitating critical interhemispheric communication. Anomalies of the CC, ranging from complete agenesis (ACC) to hypoplasia and dysgenesis, are frequent manifestations of complex neurodevelopmental syndromes. While mouse has historically served as the primary model for these disorders due to its conserved mammalian neuroanatomy, zebrafish has emerged as a powerful, albeit non-mammalian, alternative. This review synthesizes data from human, mouse, and zebrafish genetic tables to highlight the anomalies occurring at distinct steps of CC development. We examine how mouse models have been instrumental in mapping the structural failures of commissure formation. Furthermore, we address the utility of zebrafish models demonstrating that they effectively model the underlying cellular mechanisms of these disorders. Through an analysis of convergent phenotypes and divergent phenotypic readouts, we state that zebrafish provide a complementary platform for dissecting the molecular etiology of human callosal disorders.

## Introduction

The integration of sensory, motor, and cognitive functions between the two cerebral hemispheres relies on the CC, a massive tract consisting of over 200 million axons in humans (Wise, 1975; Zhou et al., 2013). Because of its developmental complexity, the CC is highly susceptible to genetic perturbations. Agenesis of the Corpus Callosum (ACC) is among the most common brain malformations, occurring in approximately 1 in 4,000 live births and often associated with intellectual disability, seizures, and social deficits (Hofman et al., 2020). To understand the etiology of these defects, researchers rely on model organisms to recapitulate human syndromes. We have categorized the genetic landscape of these disorders into three distinct biological contexts: human syndromes associated with forebrain commissural defects, mouse models utilized for conditional knockouts, and zebrafish models. While mouse shares the fundamental commissural architecture with humans, making it ideal for studying gross anatomical defects, zebrafish offers unique advantages in visualizing early cellular processes. Despite lacking a definitive corpus callosum, zebrafish possess homologous commissural tracts (e.g., anterior and post-optic commissures), and the molecular machinery driving axon guidance is deeply conserved across vertebrates (Culverwell and Karlstrom, 2002; Varela-Echavarría and Guthrie, 1997). Consequently, genes responsible for human CC defects often manifest as axonal tract disruptions or generalized axonopathies in zebrafish, revealing “mechanistic conservation” amidst “anatomical divergence.” This review highlights the developmental steps prone to error from midline patterning to axonal extension and evaluates the conservation of gene function across species. It specifically addresses the paradox of using the acallosal zebrafish to study callosal disorders, revealing how deep genetic conservation allows for the modeling of core neurodevelopmental mechanisms despite anatomical divergence.

## Telencephalic commissural anomalies of the brain

The proper functioning of a bilateral brain relies heavily on the coordination between the two cerebral hemispheres, facilitated by tracts of nerve fibers known as commissures (Gazzaniga, 2000). These pathways are critical for maintaining functional coordination and integrating motor, sensory, and cognitive processes, such as memory. The major commissural routes include the habenular, posterior, anterior, hippocampal, and corpus callosum (Gazzaniga, 2000; Ocklenburg and Guo, 2024). The development of these structures is a complex, multi-step process. It begins with patterning, cellular proliferation, and specification, followed by neuronal and glial interactions, responses to axonal guidance cues, growth, migration, and the establishment of contralateral connectivity (Paul et al., 2007). Disruption at any stage of this developmental timeline results in agenesis or dysgenesis of the commissures (Minocha and Herr, 2019; Paul, 2011).

### Corpus callosum and its anomalies

The CC is the largest commissure, consisting of approximately 190 million heavily myelinated axons that primarily create homotopic or heterotopic projections between the hemispheres (Tanaka-Arakawa et al., 2015). Its development begins around the 10th gestational week with the formation of the *massa commissuralis* and then keeps growing, thickening, and maturing all the way into young adulthood (Tole et al., 2006). While the CC is fully developed by age four, it continues to grow at a slower rate until the thirties. It is an integrative conduit for transmitting motor, sensory and cognitive impulses between the cerebral hemispheres (Paul et al., 2007). Structural defects of the CC are among the most frequent brain anomalies observed at birth, despite being uncommonly prevalent in the general population. ACC is a rare anomaly, occurring in about 1.85–2.49 per 10,000 births, yet it remains the most common form of callosal defect (Castellani, 2013; Edwards et al., 2014; Paul, 2011). Studies suggest that men are more likely to develop CC abnormalities than women (Glass et al., 2008). ACC is morphologically categorized into two types: Type 1, where axons fail to cross the midline and form a Probst bundle, and Type 2, where the commissural axons fail to form entirely (Glass et al., 2008). Other defects include hypoplasia (thinning) and hyperplasia (thickening) (Leombroni et al., 2018; Supplementary Table 1).

The etiology of these defects is multifactorial and includes genetic, environmental, metabolic, infectious, and vascular factors. Prenatal exposure to alcohol has been reported to disrupt signaling and hamper inter-hemispheral connectivity (Mira et al., 2020). Similarly, smoking and cannabis consumption pose risks for developmental defects (Ruisch et al., 2018). Environmental pollutants, specifically PM2.5 levels during the prenatal period, have been linked to CC thinning and behavioral anomalies (Mortamais et al., 2019). Metabolic conditions also play a role; for example, mothers with untreated Phenylketonuria (PKU) are known to produce offspring with CC hypoplasia due to reduced myelination (Levy et al., 1996). A substantial proportion of callosal malformations are associated with genetic etiologies, including monogenic mutations affecting axon guidance, midline patterning, and cytoskeletal regulation, contributing to failed commissural crossing during embryogenesis (Briançon-Marjollet et al., 2008; Brudvig et al., 2018; Gonda et al., 2020; Leombroni et al., 2018; Marsh et al., 2017; McConnell et al., 2016). Additionally, acquired prenatal factors such as TORCH infections, fetal vascular insults, and maternal diabetes can also severely impair normal commissural development (Dhakal et al., 2024; Hashami et al., 2010; Lucignani et al., 2022; Russ et al., 2025).

## Molecular and cellular mechanisms of commissure development and associated anomalies

### Midline patterning and glial control

A critical prerequisite for commissure formation is the correct patterning of the midline, regulated by three glial populations: the glial wedge (GW), midline zipper glia (MG), and indusium griseum glia (IGG). The GW, located at the cortico-septal boundary, forms a substrate for callosal axons projecting toward the midline (Pescatori et al., 2017). IGG is present toward the cortical or dorsal surface, and MG lies toward the ventral surface of the CC, respectively. The IGG and MG express Slit2 and coordinated signaling through Netrin/DCC and Semaphorin pathways to integrate attractive and repulsive cues to fine-tune callosal axon trajectory (Brudvig et al., 2018; Marìn et al., 2001). Genetic regulation of these glial cells is paramount. The nuclear factor 1 (NF1) family of transcription factors, specifically *NF1A* and *NF1B*, are essential for the maturation of midline glia (Lindwall et al., 2007; Piper et al., 2009; Senaratne and Quintero-Rivera, 1993). Loss of *NF1A* and *NFIB* disrupts the essential radial glia-to-astroglia conversion needed for interhemispheric fissure remodeling, creating a mechanical barrier that physically blocks midline axonal crossing (Piper et al., 2009; Senaratne and Quintero-Rivera, 1993). Midline glial specification is further regulated by morphogen gradients, including Sonic hedgehog (Shh), BMP, FGF and Wnt signaling pathways, and ciliary proteins, which establish dorsoventral identity and ensure appropriate commissural substrate formation (Dafinger et al., 2011; Greig et al., 2013; Keeble et al., 2006). This links commissural defects to ciliopathies like Joubert syndrome, which involves mutations in genes such as *KIF7*, *CEP290*, NSMF, NPHP1, and *TMEM67* (Dafinger et al., 2011; Doherty et al., 2010; O’Driscoll et al., 2010; Parisi, 2009; Poretti et al., 2011). The temporal synchronization between midline glial maturation and the arrival of pioneer callosal axons is critical, as premature or delayed glial differentiation can disrupt commissural scaffold formation (Jovanov-Milosevic, 2009; Lynton et al., 2023; Shu et al., 2003; Silver and Ogawa, 1983). Collectively, these findings underscore that commissure formation represents a tightly coordinated neuro-glial developmental program rather than solely an axonal event.

### Neuronal specification and migration

**FIGURE 1:**
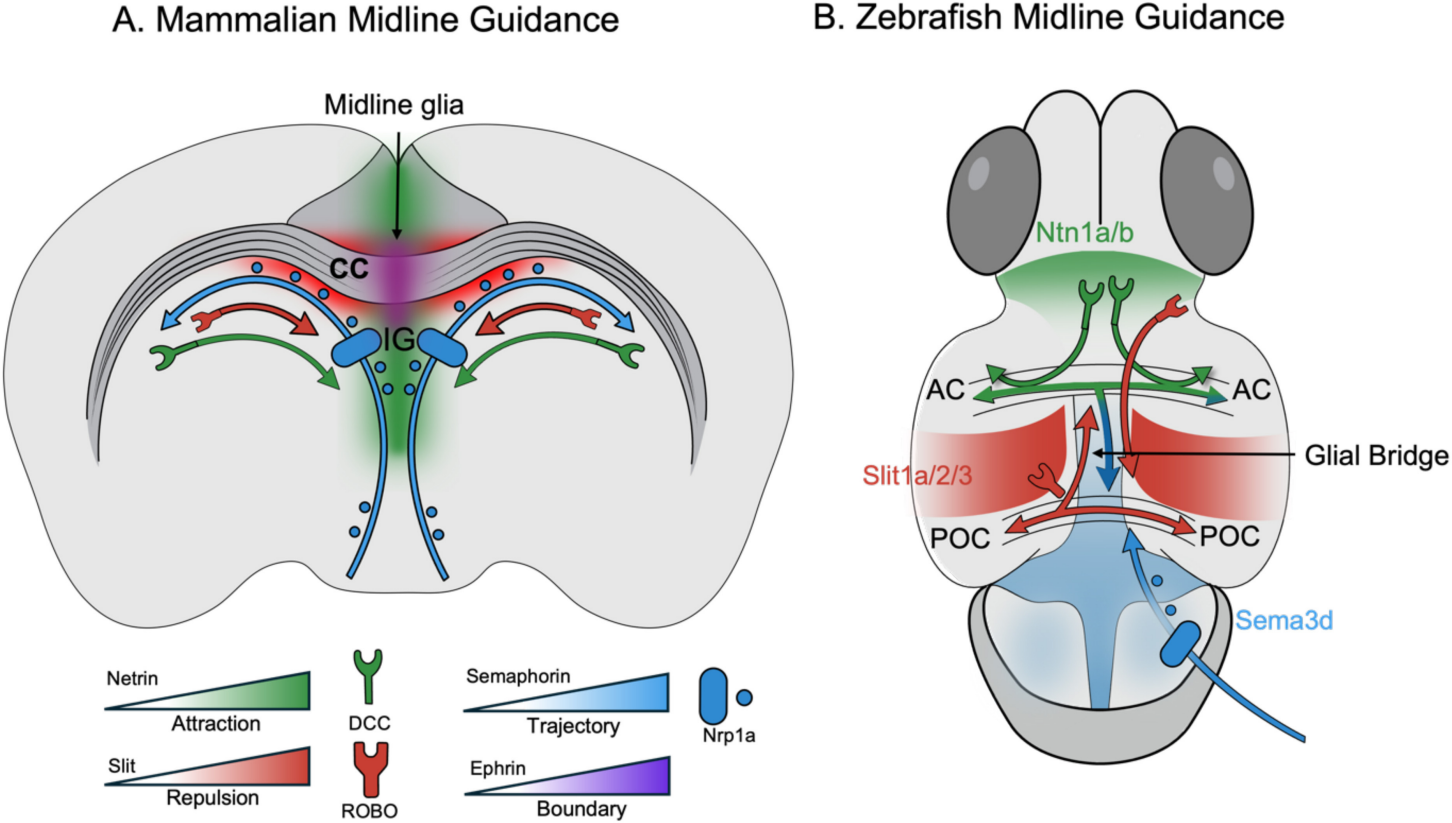
Mechanistic conservation of midline axon guidance cues in mammalian and zebrafish forebrains. **(A)** In the mammalian forebrain, midline glia and structures like the Indusium Griseum (IG) orchestrate the formation of the Corpus Callosum (CC). A gradient of Netrin (green) attracts pioneer axons expressing the DCC receptor toward the midline. Conversely, Slit (red) provides a repulsive boundary, preventing ROBO-expressing axons from re-crossing. Semaphorins (blue) interact with Nrp1a receptors to fine-tune axonal trajectory, while Ephrins (purple) help establish structural boundaries. **(B)** Although zebrafish lack a corpus callosum, the developing forebrain relies on a highly conserved suite of molecular cues to guide homologous proxy tracts, specifically the Anterior Commissure (AC) and Post-optic Commissure (POC). Netrin paralogs (Ntn1a/b) attract navigating axons toward the midline. Slit paralogs (Slit1a/2/3), secreted by the centrally located Glial Bridge, repel these axons to tightly channel them into commissural tracts. Semaphorins (e.g., Sema3d) further facilitate crossing via Nrp1a receptors. Diagram depicting the relative positioning of the anterior and post-optic commissures and associated guidance cues. Spatial relationships are simplified for clarity and do not reflect exact anatomical boundaries, and may vary across developmental stages.

The neocortex develops through the proliferation and differentiation of neuroepithelial cells, generating radial glial cells (RGCs) and intermediate progenitor cells (IPCs) which give rise to immature migrating neurons forming six layers of the cortex (Buchsbaum and Cappello, 2019; Malatesta et al., 2000; Noctor et al., 2001; Rakic, 2009; Subramanian et al., 2020). This process is tightly regulated by transcription factors that determine neuronal identity and laminar fate, including Satb2, Ctip2, and Tbr1, which distinguish callosal from subcortical projection neuron lineages (Noctor et al., 2001; Rakic, 2009; Wise, 1975). Callosal projection neurons (CPN) present in layer II-III for the cortex, migrate toward the midline, guided by a “subcallosal sling” formed by glial populations (Lindwall et al., 2007; Figure 1). Proper specification of CPN identity is essential prior to midline navigation, as fate mis-specification can redirect axons toward inappropriate subcortical targets (Leyva-Díaz and López-Bendito, 2013). Cytoskeletal remodeling coordinates nucleokinesis, leading process extension, and directional persistence during radial and tangential migration (Deuel et al., 2006; Poirier et al., 2007). Mutations in *ANKB* (Ankyrin 2), which interacts with dynactin to control axonal cargo motility, result in impaired axonal projections and partial ACC (Lorenzo et al., 2014). Intracellular trafficking mechanisms, including dynein-dynactin–mediated transport, are therefore critical for sustaining long-range commissural growth (Lorenzo et al., 2014; Prokop, 2013). Similarly, cell adhesion molecules like *CHL1* and laminins such as *LAMC1* are crucial for mediating signals from guidance receptors; their absence leads to abnormal axonal growth and migration (Demyanenko et al., 2011; Maness and Schachner, 2007). Thus, extracellular matrix cues, integrin signaling, and adhesion molecules tightly coordinate cytoskeletal regulation and neuronal specification to enable stable commissural connectivity.

### Reception of guidance cues

Axonal navigation is orchestrated by guidance cues which function in spatially and temporally regulated manner, ensuring that pioneer axons reach and cross the midline before later-arriving fibers (Paul et al., 2007). The Slit-Robo signaling pathway is a primary repulsive mechanism (Dudanova and Klein, 2013). The expression of Slit by the indusium griseum and glial wedge guides axons expressing ROBO receptors (Gonda et al., 2020). Slit signaling prevents aberrant midline re-crossing and ensures proper lateral positioning after commissural passage. This repulsion requires the interaction of ROBO receptors with the phosphoprotein MENA; loss of MENA disrupts filopodia extension and leads to disorganized commissures (McConnell et al., 2016). Actin cytoskeletal remodeling downstream of these interactions is critical for growth cone steering and directional stability (Dent et al., 2011; Forscher and Smith, 1988). Conversely, Netrin-1, signaling through the DCC receptor, acts as a crucial attractant for callosal axons (Marsh et al., 2017). The balance between Netrin-mediated attraction and Slit-mediated repulsion establishes the permissive corridor for midline crossing (Bagri et al., 2002; Serafini et al., 1996). *Netrin-1* mutations can cause severe disruption, including the complete absence of the HC and AC (Brudvig et al., 2018). Furthermore, Ephrin B1 (*EFNB1*) is essential for neurite outgrowth, with mutations located on the X chromosome leading to Cranio-frontonasal syndrome and callosal dysgenesis (Twigg et al., 2004). Eph-ephrin signaling additionally contributes to boundary formation and topographic mapping, refining interhemispheric connectivity after initial crossing (Hu et al., 2003; Liebl et al., 2003). Collectively, the integration of attractive, repulsive, and contact-mediated cues ensures the fidelity of commissural tract formation.

### Axonal navigation and connectivity

Upon reaching the midline, axons must navigate specific corridors. This navigation depends on region-specific transcriptional programs that confer positional identity to commissural neurons prior to crossing (Herrera and Escalante, 2022; Zarin et al., 2014). Axons passing through the anterior *massa commissuralis* form the CC (expressing *Emx1* and *Nfia*), while those in the posterior region form the hippocampal commissure (associated with *Six3* and *Zic2*) (Moldrich et al., 2010). The anterior commissure is formed by axons passing through the *area septalis*, expressing *Six3*. These regionally restricted transcription factors not only define neuronal identity but also regulate responsiveness to distinct guidance cues along the anterior–posterior axis. Deletions in *Zic2* (13q32.3-q33.1) are specifically linked to holoprosencephaly and cerebellar abnormalities alongside ACC (Hatayama et al., 2011; O’Driscoll et al., 2010). ZIC2 is also critical for midline patterning and forebrain division, explaining the frequent co-occurrence of commissural and prosencephalic defects. Finally, the establishment of contralateral connections involves both homotopic and heterotopic projections (Gobius et al., 2016; Yorke and Caviness, 1975; Figure 1). Activity-dependent refinement further shapes these projections postnatally to ensure functional interhemispheric integration. Transcription factors like *CREB1* regulate actin polymerization in growth cones via *Gap43* to ensure proper connectivity; loss of *CREB1* leads to hypoplasia (Kitazawa et al., 2012; Teng and Tang, 2006). This highlights the importance of intracellular signaling cascades in stabilizing axonal extension after midline crossing. Metabolic enzymes also play a role; for example, defects in cholesterol metabolism (e.g., *DHCR24*) disrupt Shh signaling, leading to abnormal axonal patterning (Zolotushko et al., 2011). Perturbation of lipid homeostasis can therefore secondarily impair morphogen signaling and growth cone responsiveness. A notable specific pathology is Autosomal Recessive Spastic Paraplegia 11, caused by *SPG11* mutations, which presents with a thin CC and a “ears of the lynx” sign on MRI (Pascual et al., 2019; Sjaastad et al., 2018; Tian et al., 2016). This underscores how defects in axonal maintenance and intracellular trafficking can lead to progressive callosal thinning. Collectively, successful commissural connectivity requires coordinated transcriptional specification, metabolic integrity, cytoskeletal regulation, and post-crossing refinement mechanisms.

## Zebrafish as a model for callosal disorders: convergent mechanisms and divergent insights

A striking finding from the comparative analysis is the strong conservation of phenotypes related to brain size and telencephalic development across all three species (Human, mouse, and zebrafish) (Cárdenas and Borrell, 2020). The shared developmental modules underscore that core mechanisms governing neurogenesis, progenitor proliferation, and axonal growth are deeply conserved across vertebrates. While mouse has long been the gold standard for modeling mammalian brain structure due to its anatomical similarities to humans (Supplementary Table 2), zebrafish has emerged as a powerful complementary model. Although zebrafish lack a corpus callosum *per se*, they possess commissural tracts and conserved midline guidance systems that recapitulate essential molecular pathways involved in commissural development (Chia et al., 2022; Suãrez et al., 2014). Zebrafish offer unique advantages, including optical transparency during embryogenesis, high fecundity, and a rapidly developing nervous system that is genetically accessible (Adamson et al., 2018; Chia et al., 2022). The availability of transgenic reporter lines and CRISPR-based genome editing alongside high-throughput genetic and pharmacological screening, facilitates rapid identification of candidate modifiers relevant to human commissural disorders. Together, these features position zebrafish as a tractable system for dissecting conserved molecular mechanisms while providing divergent evolutionary insights into forebrain connectivity.

## Conservation of core neurodevelopmental phenotypes

The profound cross-species conservation of brain size and forebrain development phenotypes underscore the ancient evolutionary origins of the genes governing cell division and cytoskeletal architecture.

The gene *WDR62* (WD Repeat Domain 62) exemplifies this deep conservation. In humans, mutations in *WDR62* cause Microcephaly 2, a condition characterized by a significantly reduced brain size, cortical malformations, and pachygyria (Bilgüvar et al., 2010; Yu et al., 2010). Similarly, mouse models deficient in *Wdr62* exhibit microcephaly and cortical thickening (Bilgüvar et al., 2010; Nicholas et al., 2010). Crucially, zebrafish models recapitulate this core phenotype, displaying a marked reduction in brain size (microcephaly) (Novorol et al., 2013). This phenotypic similarity across species validates zebrafish as a robust model for studying the ancient genetic mechanisms underlying human brain development and telencephalic malformations.

Similarly, *ASPM* (Abnormal Spindle Microcephaly Associated), the most common cause of primary microcephaly in humans, shows conserved phenotypic effects (Bond et al., 2002; Pattison et al., 2000). Human patients present with simplified gyri and hypoplastic cerebellums, while zebrafish models mirror the reduction in overall brain size (Novorol et al., 2013). The conservation extends to cytoskeletal genes like *TUBA1A* (Tubulin Alpha 1a). In humans, *TUBA1A* mutations lead to Lissencephaly type 3, a severe “smooth brain” phenotype associated with CC dysgenesis (Poirier et al., 2007). Zebrafish models reveal “lissencephaly-like phenotypes,” characterized by motor axonopathy and reduced neurite outgrowth (Veldman et al., 2010). Disruptions in deeply conserved genes governing progenitor proliferation and cytoskeletal integrity cause both structural anomalies such as microcephaly and callosal dysgenesis and functional circuit deficits (Bartoszewski et al., 2022). While uniquely mammalian features like gyrification require mammalian models, this evolutionary conservation makes zebrafish a highly effective complementary model for investigating the fundamental mechanisms of brain malformations (Table 1).

**TABLE 1:** Zebrafish Models of Human brain disorders focusing on developmental and axonal tract defects.

Gene name	Gene name	Chromosomal location	Zebrafish defects	Associated human disorder(s)	References
Aldolase, fructose-bisphosphate	*Adolaa*	Chr3	Narrowed forebrain, reduced and disorganized forebrain and hindbrain axon tracts. Defective touch response (no response). Decreased neural progenitor formation	16p11.2 deletion Syndrome	Blaker-Lee et al. (2012)
Autophagy related 16 like 1	*Atg16l1*	Chr2	Impaired lens autophagy (Cataract like)	Alzheimer’s diseases	Cui et al. (2024)
Abnormal spindle-like, microcephaly-associated	*Aspm*	Chr1	Microcephaly (reduced brain size)	Microcephaly	Novorol et al. (2013)
CDK5 and ABL1 enzyme substrate 1	*Cables*	Chr2	Necrosis in midbrain and hindbrain, Neural tube patterning defects	Cushing Syndrome	Groeneweg et al. (2011)
Collapsin response mediator protein 2	*Crmp2*	Chr10	Reduction of axon bundles in the anterior commissure and postoptic commissure	Alzheimer’s diease, bipolar disorder, schizophrenia, epilepsy	Guo et al. (2021)
Collapsin response mediator protein 4	*Crmp4*	Chr4	Reduction of axon bundles in the anterior commissure and postoptic commissure	Amyotrophic Lateral Sclerosis (ALS)	Guo et al. (2021)
Disrupted in schizophrenia 1	*Disc1*	Chr13	Defects in forebrain formation and failure of axon outgrowth throughout the brain. Reduced hindbrain and midbrain ventricles Abnormal development of hypothalamic progenitor cells (rx3++) Compromised neuronal differentiation (abnormal ff1b+ and crh+ neurons). Failure to upregulate cortisol under stress; abnormal social behavior (shoaling).	Schizophrenia, Bipolar Disorder, Major Depression	Brandon et al. (2009) and Eachus et al. (2017)
Esrom	*Esrom*	Chr9	Loss of habenular commissure	Not known	Hendricks et al. (2008)
Exostosin glycosyltransferase 2	*Ext2*	Chr1	Defects in craniofacial cartilage, axon sorting, tooth development, lack of pectoral fin	Hereditary multiple exostoses	Lee et al. (2004) and Clément et al. (2008)
Forkhead Box P2	*Foxp2*	Chr3	Defects in anterior commissure, postoptic commissure and Supraoptic tract. Increased locomotive activity.	Autism Spectrum Disorders (ASD) and Attention-Deficit/Hyperactivity Disorder (ADHD)	Lüffe et al. (2021)
Family with sequence similarity 57 member Ba	*Fam57ba*	Chr16	Small eye cups, narrowed forebrain, reduced/disorganized axon tracts	16p11.2 deletion Syndrome, ADHD, Epilepsy	Blaker-Lee et al. (2012) and McCammon et al. (2017)
Fragile X messenger ribonucleoprotein 1	*Fmr1*	Chr14	Abnormal mid-hindbrain boundary. Increased branching of trigeminal and Rohon-Beard neurites. Altered tectal coding of visual stimuli (more diffuse representations) Altered social behavior (slower response to social cues)	Fragile X Syndrome, Autism Spectrum Disorder (ASD)	Tucker et al. (2006), den Broeder et al. (2009), and Zhu et al. (2023)
Growth associated protein 43	*Gap43*	Chr15	Reduced neurite outgrowth and synaptic plasticity	Alzheimer’s diseases	Udvadia et al. (2001) and Kusik et al. (2010)
Kinesin light chain 1	*Klc1*	Chr13	Defective anterior and postoptic commissure formation	Alzheimer’s and Parkinson’s diseases	Li et al. (2024)
Kinesin family member 22	*Kif22*	Chr12	Narrowed forebrain, reduced and disorganized forebrain axon tracts. Defective touch response. Decreased neural progenitor formation	16p11.2 deletion syndrome	Blaker-Lee et al. (2012)
Potassium channel tetramerization domain containing 13	*Kctd13*	Chr17	Macrocephaly (increased head/telencephalon size) upon knockdown. Microcephaly (decreased head/telencephalon size) upon overexpression. Altered neuronal proliferation in the brain (increased in knockdown, decreased in overexpression). Reduced and disorganized forebrain axon tracts	16p11.2 deletion Syndrome, Autism Spectrum Disorder (ASD)	Golzio et al. (2012) and Blaker-Lee et al. (2012)
L1 cell adhesion molecule	*L1Cam*	Chr23	hydrocephalus, defects in axonal outgrowth, and myelination abnormalities	CRASH1 or L1 syndrome	Linneberg et al. (2019)
LIM homeobox 2	*Lhx2*	Chr8	Midline axon guidance, forebrain patterning and eye morphogenesi	Intellectual disability, developmental delay, and microphthalmia	Seth et al. (2006) and Peukert et al. (2011)
Mitogen-activated protein kinase 3	*Mapk3*	Chr3	Reduced and disorganized forebrain and hindbrain axon tracts. Suppression rescues microcephaly and neuronal proliferation phenotypes induced by KCTD13 overexpression.	16p11.2 deletion Syndrome, Schizophrenia	Blaker-Lee et al. (2012) and Gusev et al. (2018)
Neurofibromatosis type 1	*Nf1a/b*	Chr1	Glial proliferation abnormalities, hypomyelination, improper glial differentiation, learning and motor deficits, and heightened susceptibility to malignant gliomas.	Neurofibromatosis. Learning disabilities, attention deficit, motor deficits, and in some cases, intellectual impairment	Shin et al. (2012)
Nestin	*Nes*	Chr16	Small head and small eyes. Hydrocephalus and dilated brain ventricles. Reduced hindbrain and midbrain size. Unclear boundaries between brain subdivisions. Increased apoptosis of neural progenitor cells.	Neurodevelopmental disorders	Chen et al. (2010)
Outer dense fiber of sperm tails 2	*Odf2*	Chr21	Microcephaly (reduced brain size)	Microcephaly	Novorol et al. (2013)
Paired domain transcription factor 2	*Pax2*	Chr13	Altered retinal patterning and ventral forebrain defect. Coloboma	Renal Coloboma Syndrome	Bertuzzi et al. (1999)
Proteasome 26S subunit, non-ATPase 12	*Psmd12*	Chr11	Microcephaly (significantly smaller optic tecta).	Neurodevelopmental disorders with Intellectual disability and autism spectrum disorder (ASD)	Küry et al. (2017)
Rab small GTPases	*Rab33a; Rab33ba*	Chr14	dysgensis anterior and postoptic commissure formation	Smith–McCort dysplasia	Huang et al. (2019)
Ras homolog, mTORC1 binding	*Rheb*	Chr24	Increased head size. Aberrant neuronal migration. Induction of seizures	Megalencephaly, Epilepsy	Reijnders et al. (2017)
Spastic paraplegia 11	*Spg11 (Kiaa1840)*	Chr25	Mild hydrocephaly, the mid-hindbrain boundary was reduced and small eyes. Shorter cranial motor neurons.	Hereditary spastic paraplegia with thin corpus callosum	Southgate et al. (2010)
Stem loop binding protein	*Slbp*	Chr14	Dent in the midbrain-hindbrain boundary (MHB), Coloboma	Not known	Turner et al. (2019)
SNARE associated protein 29	*Snap29*	Chr8	Defective trigeminal nerve formation and excess axonal branching. swimming difficulties.	CEDNIK syndrome (Cerebral Dysgenesis, Neuropathy, Ichthyosis and Keratoderma)	Mastrodonato et al. (2019)
SCL/TAL1 interrupting locus	*Stil*	Chr22	Microcephaly (reduced brain size)	Microcephaly	Novorol et al. (2013)
Syntaxin 1b	*Syngap1b*	Chr19	Delayed mid- and hindbrain development. Extensive neuronal cell death in the embryonic nervous system. Spontaneous, seizure-like behaviors	Phelan-McDermid Syndrome, Autism Spectrum Disorder (ASD), Schizophrenia	Kozol et al. (2015)
Alpha-tubulin 1A	*Tuba1a*	Chr23	Motor axonopathy, reduced neurite outgrowth, and brain malformations (e.g., lissencephaly-like phenotypes)	Lissencephaly	Veldman et al. (2010).
Trio rho guanine nucleotide exchange factor	*Trio*	Chr1	Neural crest cell (NCC) migration deficits, leading to mandibular retrusion and craniofacial abnormalities	Intellectual Disability, Sutism Spectrum Disorder, and other developmental delays.	Guo et al. (2021) and Klems et al. (2020)
WD REPEAT PROTEIn 62	*Wdr62*	Chr15	Microcephaly (reduced brain size)	Microcephaly	Novorol et al. (2013)

## Anatomical divergence and mechanistic conservation

A major point of anatomical divergence is the absence of a corpus callosum in teleost. zebrafish brain utilizes different commissural tracts (e.g., the anterior and post-optic commissures) to connect hemispheres. However, the molecular mechanisms guiding axons across the midline are highly conserved (Liu et al., 2009; Figure 1). This allows zebrafish to model the cellular causes of human CC defects, even if the specific structure is missing. To explicitly translate the concept of mechanistic conservation into concrete experimental terms, Supplementary Table 3 maps the sequential steps of mammalian CC development to their corresponding zebrafish proxy structures, phenotypic readouts, and experimental assays.

For instance, the *L1CAM* gene is associated with L1 Syndrome (CRASH/MASA) in humans, causing hydrocephalus and CC agenesis (Otter et al., 2017). Zebrafish models of *L1CAM* dysfunction exhibit hydrocephalus and specific defects in axonal outgrowth (Linneberg et al., 2019). Although zebrafish lacks a CC, the failure of axons to extend and navigate correctly is the shared pathological mechanism.

This principle is further illustrated by genes like *GAP43* (Growth Associated Protein 43) and *DISC1* (Disrupted in Schizophrenia 1). In mouse models, loss of these genes results in specific agenesis of the CC due to failures in axonal growth cones (Brandon et al., 2009; Ren et al., 2016; Shen et al., 2004). In zebrafish, the phenotype manifests as reduced neurite outgrowth and abnormal hypothalamic development (Eachus et al., 2017; Eachus et al., 2022; Kusik et al., 2010; Udvadia et al., 2001). Thus, the divergence between species is primarily anatomical, whereas the underlying cellular pathology remains conserved (Suãrez et al., 2014). In mice, disruption of axon growth regulators manifests as ACC; in zebrafish, the same molecular perturbations produce broad defects in neurite extension, midline crossing, or commissural tract organization (Roig-Puiggros et al., 2020). Crucially, the disruption of mammalian ACC-associated genes in zebrafish will not necessarily lead to a localized, CC-like phenotype (Suãrez et al., 2014). Instead, due to anatomical divergence, these genetic perturbations frequently present as a broader spectrum of developmental errors, including commissural tract defects, neurite outgrowth defects, brain size phenotypes, or severe disruptions in cell proliferation (Ochenkowska et al., 2022; Roig-Puiggros et al., 2020). This reflects a functional homology, in which conserved molecular disruptions generate species-specific structural outcomes while preserving a shared cellular mechanism. This reflects a functional homology, in which conserved molecular disruptions generate species-specific structural outcomes while preserving a shared cellular mechanism. This suggests that zebrafish can effectively screen for genes involved in axon guidance disorders, identifying candidates that can be further validated in mammalian models for specific CC defects.

## Pleiotropy and unique insights from zebrafish

When utilizing zebrafish to model CC anomalies, it is crucial to distinguish between phenotype classes that are directly informative for CC-associated mechanisms and those that represent divergent pleiotropic outcomes. Informative phenotypes are those that directly reflect conserved cellular processes required for commissure formation, such as disruptions in anterior or post-optic commissure development, failures in midline axon crossing, or generalized deficits in neurite outgrowth (Roig-Puiggros et al., 2020; Suãrez et al., 2014). Conversely, non-specific pleiotropic outcomes such as craniofacial abnormalities or lens defects arise because these genes often govern fundamental cellular processes across multiple tissue types (Cui et al., 2024; Guo et al., 2021). While these pleiotropic phenotypes do not directly model CC malformations, they remain highly valuable for uncovering the systemic, organism-wide functions of the implicated genes (Crouzier et al., 2021; Haynes et al., 2022). Furthermore, the optical transparency of the zebrafish allows for the observation of non-neural phenotypes that might be subtle or overlooked in rodent models.

The gene *ATG16L1*, a key autophagy regulator, provides a compelling example. In mice, heterozygous knockout leads to structural brain defects like the thickening of the CC. In contrast, zebrafish models highlight a “cataract-like” phenotype characterized by impaired lens autophagy (Cui et al., 2024). This divergence directs researchers to investigate the role of the gene in different tissues, suggesting that *ATG16L1* is critical for maintaining cellular homeostasis in both the brain (mouse) and the eye (zebrafish) via conserved autophagic pathways (Cui et al., 2024; Kannan et al., 2017).

Furthermore, zebrafish are increasingly valuable for modeling behavioral aspects of neurodevelopmental disorders. *FMR1*, the gene responsible for Fragile X Syndrome, causes structural malformations in the human mesencephalon and cerebellum (Tucker et al., 2006). While zebrafish show some structural issues (e.g., abnormal mid-hindbrain boundaries), their most significant contribution is in modeling the behavioral deficits. *FMR1*-deficient zebrafish exhibit altered social behaviors, such as a slower response to social cues and diffuse processing of visual stimuli (den Broeder et al., 2009; Zhu et al., 2023). This distinct phenotype offers a high-throughput platform for screening potential pharmacological interventions for the behavioral symptoms of autism spectrum disorders, a capability that is more labor-intensive in rodent models.

Finally, the gene *TRIO* illustrates how zebrafish can link disparate clinical features. In mice, *TRIO* loss leads to reduced CC thickness (Briançon-Marjollet et al., 2008). In zebrafish, it causes migration deficits in neural crest cells (NCC), leading to mandibular retrusion and craniofacial abnormalities (Guo et al., 2021). Collectively, these examples demonstrate that zebrafish models do not merely replicate mammalian phenotypes but instead uncover the pleiotropic cellular programs underlying neurodevelopmental genes. Through tissue-level visualization, behavioral quantification, and live developmental imaging, zebrafish expand mechanistic interpretation from isolated structural abnormalities to integrated, organism-wide developmental processes.

## Therapeutic modeling and limitations across species

The identification of conserved molecular mechanisms across human, mouse, and zebrafish models has direct translational implications for CC disorders. While anatomical outcomes vary, underlying cellular defects such as altered cytoskeletal dynamics, autophagy, and axon guidance are shared across species. Zebrafish are highly advantageous for translational research, offering rapid external development, optical transparency, and genetic tractability for high-throughput pharmacological screening enables real-time visualization of axonal pathfinding and midline crossing *in vivo* (Adamson et al., 2018; Chia et al., 2022). This enables efficient *in vivo* identification of compounds that rescue neurite extension defects, restore commissural tract organization, or modulate progenitor proliferation. Integrating zebrafish for rapid drug discovery with mammalian models for structural validation creates a powerful, tiered translational pipeline. Ultimately, this cross-species approach accelerates precision medicine by linking specific genetic disruptions to targeted interventions.

While zebrafish are exceptional for high-throughput screening and *in vivo* imaging, their utility is limited by the absence of a corpus callosum and a six-layered neocortex, precluding the study of higher-order features like cortical gyrification and laminar-specific projections. Although zebrafish lack a corpus callosum and cannot directly model the anatomical endpoints of CC-related disorders (Haynes et al., 2022), they serve as a valuable complementary system for investigating conserved developmental mechanisms. It is, therefore, critical to interpret zebrafish phenotypes at the level of underlying cellular and molecular processes, rather than as direct structural equivalents of mammalian callosal abnormalities. Accordingly, zebrafish are best utilized to analyze conserved pathways, such as axon guidance, neuronal migration, and midline patterning, that, when disrupted in mammals, lead to CC defects (Pansera et al., 2025; Suãrez et al., 2014). Additionally, teleost-specific whole-genome duplication complicates genotype-phenotype interpretations due to compensatory gene paralogs, and their behavioral assays reflect simplified circuits rather than complex human cognitive traits (Tasnim et al., 2024).

Conversely, mouse models accurately reflect mammalian callosal anatomy but are poorly suited for rapid, large-scale genetic screening. Because no single organism captures the full spectrum of human commissural anomalies, an integrative multi-species framework is essential (Haynes et al., 2022). Recognizing these limitations reinforces the importance of an integrative, multi-species approach. Mechanistic discovery in zebrafish, structural validation in mouse, and clinical correlation in humans together provide a complementary framework for understanding and ultimately addressing commissural disorders.

## Discussion

The comparative analysis of the Human, Mouse, and Zebrafish datasets illustrates that the study of corpus callosum anomalies requires a multi-model approach. Mouse models serve as the gold standard to anatomically validate specific structural defects such as agenesis or Probst bundles (Meoded et al., 2015; Moon, 2006; Popko, 1999; Winter, 1988) and mechanistically confirm midline guidance failures, as demonstrated by CABLES1 and HESX1 mutations (Dattani et al., 1998; Mizuno et al., 2014). Such structural validation remains indispensable for establishing causality in disorders defined by interhemispheric connectivity.

However, the zebrafish data reveals that anatomical homology is not a prerequisite for functional utility. Despite gross anatomical differences, zebrafish preserve the core cellular and neurodevelopmental logic of commissural formation (Pansera et al., 2025), demonstrating that callosal malformations stem from disruptions in fundamental, deeply conserved pathways, such as proliferation, neuronal migration, cytoskeletal remodeling, and axon extension highlights that commissural malformations originate from disruptions in core neurodevelopmental modules rather than from defects unique to a mammalian structure (Ochenkowska et al., 2022).

Callosal anomalies are not isolated midline defects but structural manifestations of broader systemic disruptions in pathways like centrosomal dynamics and metabolic regulation (Edwards et al., 2014; Hofman et al., 2020; Tang et al., 2009). The frequent co-occurrence of callosal agenesis with cortical malformations, craniofacial abnormalities, and neurobehavioral phenotypes in human syndromes supports this systems-level interpretation. Thus, ACC represents the structural manifestation of earlier perturbations in integrated developmental pathways.

Zebrafish provide a rapid *in vivo* platform for mechanistic discovery and screening, while mouse models offer essential structural and circuit-level validation within a mammalian brain architecture (Crouzier et al., 2021). Ultimately, by shifting the focus from strict anatomical equivalence to conserved molecular programs, this cross-species approach provides profound mechanistic insights into the shared neurodevelopmental and evolutionary foundations of human CC disorders (Hassani Nia and Wittamer, 2025; Haynes et al., 2022). Ultimately, bridging the anatomical gap across species reframes the central question from whether an organism possesses a corpus callosum to whether it preserves the molecular programs required for commissural connectivity. The evidence synthesized here supports the latter. By prioritizing conserved developmental processes over strict anatomical equivalence, a multi-species framework yields deeper mechanistic insight into CC defects and strengthens the broader understanding of vertebrate forebrain evolution and neurodevelopmental pathology.
