# Supplementary material for: Bridging the anatomical gap: evolutionary conservation of genetic mechanisms in corpus callosum disorders across human, mouse, and zebrafish
Source: Front Mol Neurosci. 2026 May 7;19:1823713. doi: 10.3389/fnmol.2026.1823713 (PMC13190390; doi:10.3389/fnmol.2026.1823713)
Supplement: Supplementary file 3 [file Table_3.docx]

Supp. Table 3: Comparative Mapping of Commissural Development in mammals and zebrafish

| **Mammalian CC formation** | **Associated Callosal Pathology** | **Zebrafish Analogous Tract** | **Zebrafish Phenotypic Readout** | **Screening Assays** |
| --- | --- | --- | --- | --- |
| **Midline Patterning & Glial Scaffolding** | ACC Type 1, Probst bundle formation | Midline glial populations, forebrain boundary formation | Midline crossing failure, disorganized glial scaffold | Whole-mount immunostaining (e.g., GFAP), *in vivo* imaging of transgenic glial reporter lines |
| **Neuronal Specification & Migration** | CC hypoplasia, misrouting of projection neurons | Telencephalic projection neurons | Migration deficits, ectopic neuronal positioning | High-throughput morphological screening, whole-mount immunostaining |
| **Reception of Guidance Cues** | Agenesis or dysgenesis of CC | Anterior commissure (AC), post-optic commissure (POC) | Misrouted axons, wandering axons, uncrossed commissures | Axonal tract tracing (e.g., DiI), whole-mount immunostaining (e.g., acetylated tubulin) |
| **Axonal Navigation & Connectivity** | Severe agenesis or failure of commissural connectivity | Contralateral forebrain projections | Severe reduction or disorganization of commissural projections | Live imaging in optically transparent embryos, confocal microscopy |
